# Assembly Processes under Severe Abiotic Filtering: Adaptation Mechanisms of Weed Vegetation to the Gradient of Soil Constraints

**DOI:** 10.1371/journal.pone.0114290

**Published:** 2014-12-04

**Authors:** Nina Nikolic, Reinhard Böcker, Ljiljana Kostic-Kravljanac, Miroslav Nikolic

**Affiliations:** 1 Institute for Multidisciplinary Research, University of Belgrade, Belgrade, Serbia; 2 Landscape Ecology and Vegetation Science (320a), Hohenheim University, Stuttgart, Germany; Agroecological Institute, China

## Abstract

**Questions:**

Effects of soil on vegetation patterns are commonly obscured by other environmental factors; clear and general relationships are difficult to find. How would community assembly processes be affected by a substantial change in soil characteristics when all other relevant factors are held constant? In particular, can we identify some functional adaptations which would underpin such soil-induced vegetation response?

**Location:**

Eastern Serbia: fields partially damaged by long-term and large-scale fluvial deposition of sulphidic waste from a Cu mine; subcontinental/submediterranean climate.

**Methods:**

We analysed the multivariate response of cereal weed assemblages (including biomass and foliar analyses) to a strong man-made soil gradient (from highly calcareous to highly acidic, nutrient-poor soils) over short distances (field scale).

**Results:**

The soil gradient favoured a substitution of calcicoles by calcifuges, and an increase in abundance of pseudometallophytes, with preferences for Atlantic climate, broad geographical distribution, hemicryptophytic life form, adapted to low-nutrient and acidic soils, with lower concentrations of Ca, and very narrow range of Cu concentrations in leaves. The trends of abundance of the different ecological groups of indicator species along the soil gradient were systematically reflected in the maintenance of leaf P concentrations, and strong homeostasis in biomass N:P ratio.

**Conclusion:**

Using annual weed vegetation at the field scale as a fairly simple model, we demonstrated links between gradients in soil properties (pH, nutrient availability) and floristic composition that are normally encountered over large geographic distances. We showed that leaf nutrient status, in particular the maintenance of leaf P concentrations and strong homeostasis of biomass N:P ratio, underpinned a clear functional response of vegetation to mineral stress. These findings can help to understand assembly processes leading to unusual, novel combinations of species which are typically observed as a consequence of strong environmental filtering, as for instance on sites affected by industrial activities.

## Introduction

Understanding of species responses to well-defined environmental gradients is fundamental to developing and testing ecological theory, improving methods of community analysis and use of indicator species in environmental assessments, and predicting the impacts of global change on vegetation [1]. Many environmental factors are confounded in comparisons of widely different geographic areas; it has, for instance, not been possible to disentangle the effect of soil from the complex effect of climate on the calcifuge-calcicole gradient in European vegetation, not even in the well-studied weed assemblages [2]–[3]. Overall, community ecology has had little success in revealing general ecological principles, in particular in explaining why communities change in a systematic way across space; this is bringing about a shift of research focus towards explicit environmental gradients and ecophysiological plant traits [4].

Compared to other types of spontaneous vegetation, annual weed vegetation is a fairly simple and predictable system, more likely to reveal general assembly rules and the relationship between plant traits and the environment [5], [6]. Habitat filtering selects for specific plant adaptations which underlay the assembly of species into local communities [7]. Filtering effects of severe abiotic constraints, as for instance under harsh soil conditions, increases the chance of observing clear and consistent associations of plant ecophysiological adaptations and environmental conditions, which are maintained irrespectively of the species involved. Leaf nutrient concentrations, however, though having a high potential to indicate habitat preferences, nutrient limitation and biogeochemical cycling [8], [9], are extremely rarely included in ecological studies with a functional trait approach. Recently, harsh and unnatural conditions at “accidental experiment” sites are being recognized as uniquely suited to provide fundamental insights into ecological processes [10]. In particular, landscapes degraded by mining activities have earlier been indicated to provide an exciting opportunity to understand some important ecological principles, which might not be apparent under natural conditions [11]. A remarkable feature of spontaneous vegetation developing on post-mining land is the occurrence of new, surprising combinations of species that bear little resemblance to the assemblages on non-affected soils [11], [12]. Although this has broadly been recognized in the emerging concept of “novel ecosystems” which arise as a consequence of anthropogenic degradation [13]-[15], little is known about adaptation mechanisms that underpin this phenomenon.

We present the results of a survey undertaken on an exceptional model locality in Eastern Serbia, where large-scale and long-term fluvial deposition of pyrite-rich tailings from a copper (Cu) mine has caused a remarkable change of alluvial soils from the unaffected calcareous, towards the acidic, nutrient-poor, Cu-enriched soils over very short distances (field scale; [16], [17]). The major research question was: how would a model system of cereal weed assemblages (characterized by species abundances, life forms, Ellenberg indicator values, chorotype and leaf element concentrations) respond to a substantial change in soil characteristics if all other factors (altitude, climate, season, year, agricultural practices, surrounding vegetation, and landscape context) are held constant. This study aimed to explore the role of several ecophysiological adaptations in explaining a response of spontaneous vegetation to a complex gradient of soil properties.

## Materials and Methods

### Research locality

Since the 1940 s, the alluvial, strongly calcareous soils in the lower course of the Timok river (Serbia) have been drastically altered by the long-term release of mining waste from the Bor copper mines (highly sulphidic copper tailings) directly into the local river system (for details see [17]). More than 10 000 ha of arable land is permanently lost for agriculture, and large additional areas have been severely degraded, most prominently at meander positions where the Timok flow slows down and creates a braided pattern of lateral river channels. The fluvial deposition of mining waste over the arable fields (via regular floods) has brought about clear and consistent gradients in soil properties and cereal crop responses along the transects perpendicular to the water channel; these gradients were strongly indicated by the concentrations of total sulphur (S_tot_) in soil [16], [17]. This pollution has created multiple constraints for plant growth: nutrient deficiency (primarily P and micronutrients), excessive concentrations of available Cu, and, as a result of the oxidative weathering of deposited pyrite, very low pH (about 4) and excessive concentrations of extractable Al. The research area is under both sub-continental and sub-Mediterranean climatic influences (Figure S1). Outside of the fluvial influence, the thermpohilous varieties of the climazonal Quercion frainetto-cerridis Rudski 1940 (1949) forests are often replaced by more xerothermic extrazonal vegetation of the Quercion pubescentis-petraeae Br.-Bl. 1931 alliance [18] because of carstic geology, dissected terrain and shallow calcareous soils on steep slopes. A major association of winter cereal weeds in the lowlands of the Timok valley has been described as *Consolida regalis-Polygonum aviculare* ass. var. lathyrethosum aphacae Kojić 1973; this therophytic community belongs to the Caucalidion R.Tx.1950 alliance and is characterized by the dominance of sub-Mediterranean and Eurasian floristic elements [19].

### Field trials

The survey included 26 privately owned fields of winter cereals (18 wheat and 8 fodder barley fields), partially damaged by waste deposition, which were located within the 5 km radius from the N 44° 0434″, E 22°31′10″ (about 70 km downstream from the pollution source). No endangered or protected species were sampled. No permission was required for this field study according to the Serbian legislation. The consent to take plant and soil samples in the cereal fields was obtained from local farmers. The fields were located in a rather narrow band between the unpolluted soils on the distal part, and severely polluted soils (where cropping had been abandoned) on the proximal part of a transect perpendicular to the river channel. A satellite image of the research area (fields belonging to the villages Rajac, Braćevac and Tamnič) is available at: http://maps.google.com/maps?ll=44.082508,22.519602andz=13andt=handhl=en); the size of the fields commonly ranged between 0.5 and 1 ha. The length of the studied transects through the partially damaged fields was on average less than 15 m. Miniscule differences in elevation along these transects were impossible to detect with the standard GPS devices (presumably the microlelevation gradient was not more than 0.5 m [16]). Herbicides are sparingly applied in this low-input system: systemics (commonly Glyphosate) are applied in the autumn before sowing, and occasionally some auxinic herbicides during the stem-elongation phase.

### Weed survey

While the selection of the fields to be surveyed was rather opportunistic, vegetation and soil sampling within each field was carried out according to the flexible systematic model, a form of stratified sampling where samples were taken on the basis of the visual symptoms in the cereal crop (Table 1). In each field, it was possible to clearly distinguish two to four (depending on the site-specific topography) physiognomically uniform areas (zones) along the transect perpendicular to the river channel, as previously described [16]. Of a total of 100 samples (relevées), 26 samples each were taken in Zones 1, 3 and 4, while Zone 2 was represented by 22 samples (plots). Because of the narrow and elongated shape of the smallholder fields surveyed (see the link to the map), the area of each relevée was selected to be a rectangle of 30 m^2^ (commonly 5 m×6 m). The internet associated W3 TROPICOS nomenclatural database of the Missouri Botanical Garden, and the associated authority files (http://www.tropicos.org/Home.aspx), were used as the reference.

**Table 1:** Visual symptoms in the cereal crops as a basis for sampling along the spatial gradient on soils affected by pyritic Cu tailings.

	Visual symptoms					
Zones	Growth reduction	Chlorosis	Translucent ears	Fungal shoot infestation	Weed infestation	Rel. yield reduction (%)
1	−	−	−	−	−	n.a.
2	−	−	+	+	+	<30
3	++	++	++	++	+++	30–70
4	+++	+++	+++	+++	+++	70–95

−, none; +, low; ++, moderate; +++, severe.

Relative yield reduction was measured *a posteriori*.

To reduce the noise and increase the chance of capturing the response of weed vegetation to soil, we have excluded vernal ephemerals and late-summer (i.e. post-harvest, stubble) species, which are normally less dependent on the soil properties, and more on the season [20]. The vegetation of the selected 26 cereal fields was continuously monitored (checked every 10 days) during the period from 15^th^ May to 14^th^ July 2009. Cover-abundance of weed species was estimated on the extended nine-grade Braun-Blanquet scale [21] at every check; the final scores used for each species were the highest recorded values during the monitoring period.

### Plant sampling and analyses

Weed shoot biomass assessments and leaf sampling were carried out at the onset of milky development of the cereal crop, immediately after anthesis (Zadoks growth stage Z71–75), when the typical weed association (Consolido-Polygonetum avicularis) is fully developed. In each sample, one 1 m×1 m quadrat was laid for destructive sampling; biomass was clipped, sorted by species, air dried and weighed. For each weed species encountered, ten leaves were taken for a composite sample for tissue elemental analyses, as well as 20 flag (youngest fully emerged) leaves from a cereal crop. Crop plants were sampled for chemical analysis only if they were alive and able to set at least four seeds in the spike.

Leaf samples were thoroughly washed with deionized water, dried at 70°C and digested with a mixture of conc. HNO_3_ and H_2_O_2_ (3∶2) in a microwave oven. Upon digestion, the concentrations of elements shown in this paper (P, S, Ca, Fe, Cu, and Al) were determined by inductively coupled plasma optical emission spectrometry (ICP-OES; SpectroGenesis EOP II, Spectro Analytical Instruments GmbH, Kleve, Germany). The concentrations of N in leaf samples were determined by a CHNOS elemental analyzer (Vario ELIII, Elementar Analysensysteme GmbH, Hanau, Germany). The certified reference material (GBW10015 Spinach; Institute for Geophysical and Geochemical Exploration, Langfang, China) was used to assess the accuracy and precision of plant analyses.

### Soil sampling and analyses

A composite soil sample was obtained by sampling a soil core (30 cm height, 6.5 cm diameter) at three locations in each weed relevée. Soil samples were analyzed in fine earth fraction (<2 mm), after drying and sieving through a 2-mm mesh screen. The pH was measured in water (soil:water = 1∶2.5), and the concentrations of total C, N and S by the CHNOS elemental analyzer. Organic carbon (C_org_) was calculated from total C and CaCO_3_. Different extraction procedures were applied to determine plant-available concentrations of elements [22]: ammonium lactate - ammonium acetate (AL) for P and K, ammonium acetate (AAc) for Mg and Ca, KCl extraction for Al, and hot water (70°C) for B. Plant-available fractions of other metals (Fe, Cu, Zn and Mn) were extracted by the DTPA-TEA solution (buffered at pH 7.3), with the soil:solution ratio of 1∶5 [23]. The concentrations of chemical elements in soil samples subjected to different extraction procedures were determined by ICP-OES, with the exception of the P concentrations, which were determined by the colorimetric molybdenum blue method at 580 nm. Potential CEC was determined by ammonium acetate extraction buffered at pH 7 (with ethanol treatment adjusted for salty and carbonate samples). The precision and accuracy of the analysis was assessed with the certified reference material (GBW07417a; Institute for Geophysical and Geochemical Exploration, Langfang, China).

### Statistical analyses

For all the subsequent analyses, the records on the nine-grade Braun-Blanquet scale were transformed to a quasi-metric 1–9 scale of ordinal transform values (OTV, [21]). Species not achieving at least 20% frequency in at least one sampling zone, and species with frequency less than 10% along the transect, were excluded from further analyses; as well as outliers flagged by more than 2.3 standard deviations from the average distance between the species in a sample space. The final set comprised 84 weed species recorded in 100 relevées (Table S4).

All multivariate analyses were done by PC-ORD version 6.15 software (MjM Software Design, Gleneden Beach, USA). Sørensen distance was used in all analyses. Only one data transformation was applied: relativization of OTV by sample unit totals. To test whether the visually distinguishable zones of crop growth disorders (as defined in Table 1), used as a basis for sampling, really reflect the significant difference in weed vegetation along the gradient, the nonparametric Multi-Response Permutation Procedure [24] was used (Table S1). Indicator Species Analysis (ISA), using relativized OTV, was done after Dufrêne & Legendre [25]. Weed species with very high indication (Indicator Value IV>30%, *P* value of the Monte Carlo test <0.01) for a certain visual zone of cereal crop disorder were further classified by agglomerative cluster analysis with the space-conserving flexible beta method (β = −0.25).

The unconstrained ordination of weed vegetation data was done by Nonmetric Multidimensional Scaling (NMS), using OTV data and Sørensen distance. A final 2-d solutions with instability <10^−5^ and final stress (Monte Carlo test for stress in relation to dimensionality) of 17.55 for unrelativized and 17.76 for relativized OTV data were selected after 98 and 68 iterations for the relativized and unrelativized data, respectively. The solutions were varimax rotated. To measure the strength of association between the species, 2×2 contingency tables of presence-absence were used. Plexus values (φ coefficient) were calculated as a standardized χ^2^ statistic for species association; Yates’ correction was applied.

Univariate analysis (ANOVA) was done by STATISTICA 6 (StatSoft Inc., Tulsa, USA). Variables with approximately lognormal distribution were log-transformed prior to analyses. A posteriori comparison of means was done by Tukey’s test, with α = 0.05. Ellenberg indicator values [26] for each plot were calculated as averages weighted by the species cover-abundance (OTV). Elemental concentrations in weed vegetation (per 1 m^2^) were calculated as an average of leaf mineral concentrations in each species, weighted by the relative contribution of species to the total plot biomass (community–weighted means). The regulatory coefficient *H* was calculated from the equation: log (leaf N:P) = 1/*H*×[log (soil N:P)]+log (b); this coefficient is a continuously variable parameter which quantifies the degree of stoichiometric homeostasis of an organism across a range of environmental supply [27].

## Results

### Floristic changes

**Figure 1:**
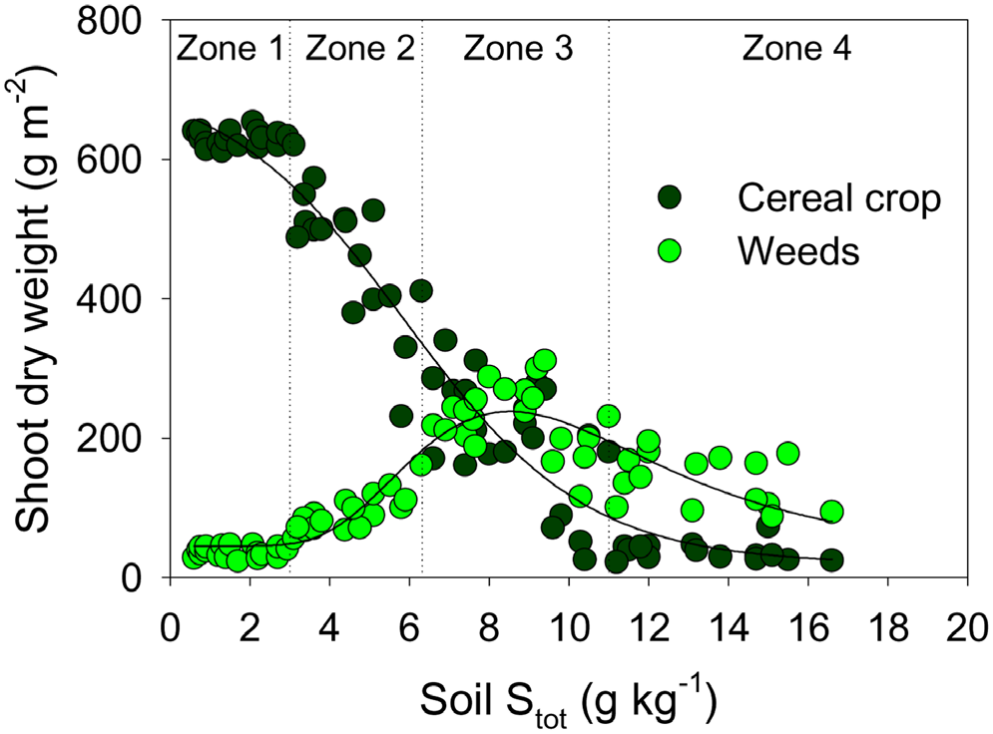
Aboveground biomass of cereal crops (milky ripeness, Z71–75) and accompanying weeds along the soil pollution gradient. Visual zones of crop growth are defined as in Table 1.

**Figure 2:**
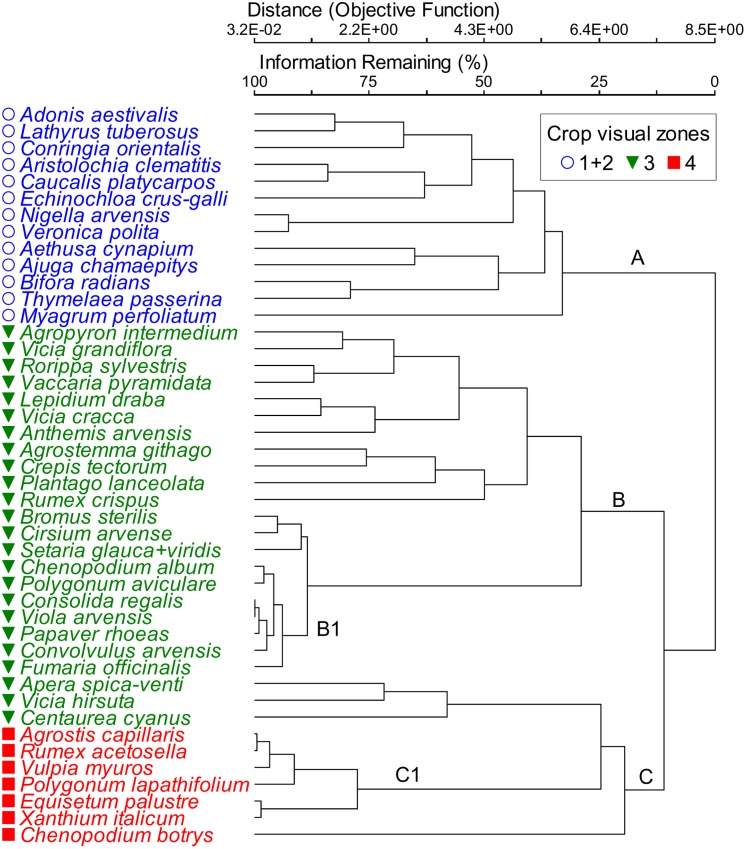
Classification of weed species that highly indicate (IV>30%, *P*<0.01) the visual zones of crop growth disorders along the soil pollution gradient. Cluster A - species of relatively unaltered calcareous soil. Cluster B - species of broad valence dominant in the middle portions of the soil gradient. Cluster C - species of most severely altered, nutrient-poor acidic soils. B1 and C1 - weeds with IV>50%, and with the major contribution to the weed biomass (measured when the crop was at milky ripeness) in Zones 3 and 4, respectively. Clustering: relative abundance, Sørensen distance, flexible linkage (β = −0.25); scaling by Wishart’s objective function.

The fluvial deposition of mining waste over arable fields caused a decrease in the total plant biomass (crop + weeds) along the transects matching the spatial gradient perpendicular to the water channel (Figure 1). While the increasing pollution load (indicated by the increasing soil S_tot_ concentrations) caused a severe growth reduction of cereal crop, the weed biomass remained at higher levels than on the relatively unaffected (S_tot_<2 g kg^−1^) soils along the gradient (Figure 1). The floristic composition of weed assemblages (recorded over the 2-months survey period) significantly varied among all the *a priori* delineated zones of crop growth disorders (Table S1). The weed assemblages on the most-severely degraded soils (Zone 4) were clearly set apart and had the highest floristic homogeneity (Table 2); the analysis of species association further showed that, along the whole transect, the φ coefficient higher than +0.5 was only found in the most severely degraded soils, namely among the following species: *Rumex acetosella*, *Agrostis capillaris*, and *Persicaria lapathifolia* (see Cluster C1, Figure 2). The differentiation of weed vegetation along the soil gradient was chiefly achieved by a remarkable shift in dominance relations (species abundances), and not by a prominent presence/absence turnover. Of the total of 84 weed species recorded in the survey, 63% were present on both relatively undamaged calcareous soils of the Zone 1 and on the most severely altered acidic soils where substantial reduction of crop growth occurs in Zone 4 (Table S2).

**Table 2:** Floristic homogeneity and species richness of weed samples in different field zones along the soil pollution gradient.

	Visual zones in polluted cereal fields			
	Zone 1	Zone 2	Zone 3	Zone 4
Weighted mean distance (relative Sørensen)	0.70	0.64	0.60	0.54
Average species number per sample	27.1	36	35.2	10.9
Total number of samples	26	22	26	26
Total species number recorded^a^	76	80	81	61

a during the 2-month survey.

Maximal abundance of all the species recorded during the survey is used.

**Figure 3:**
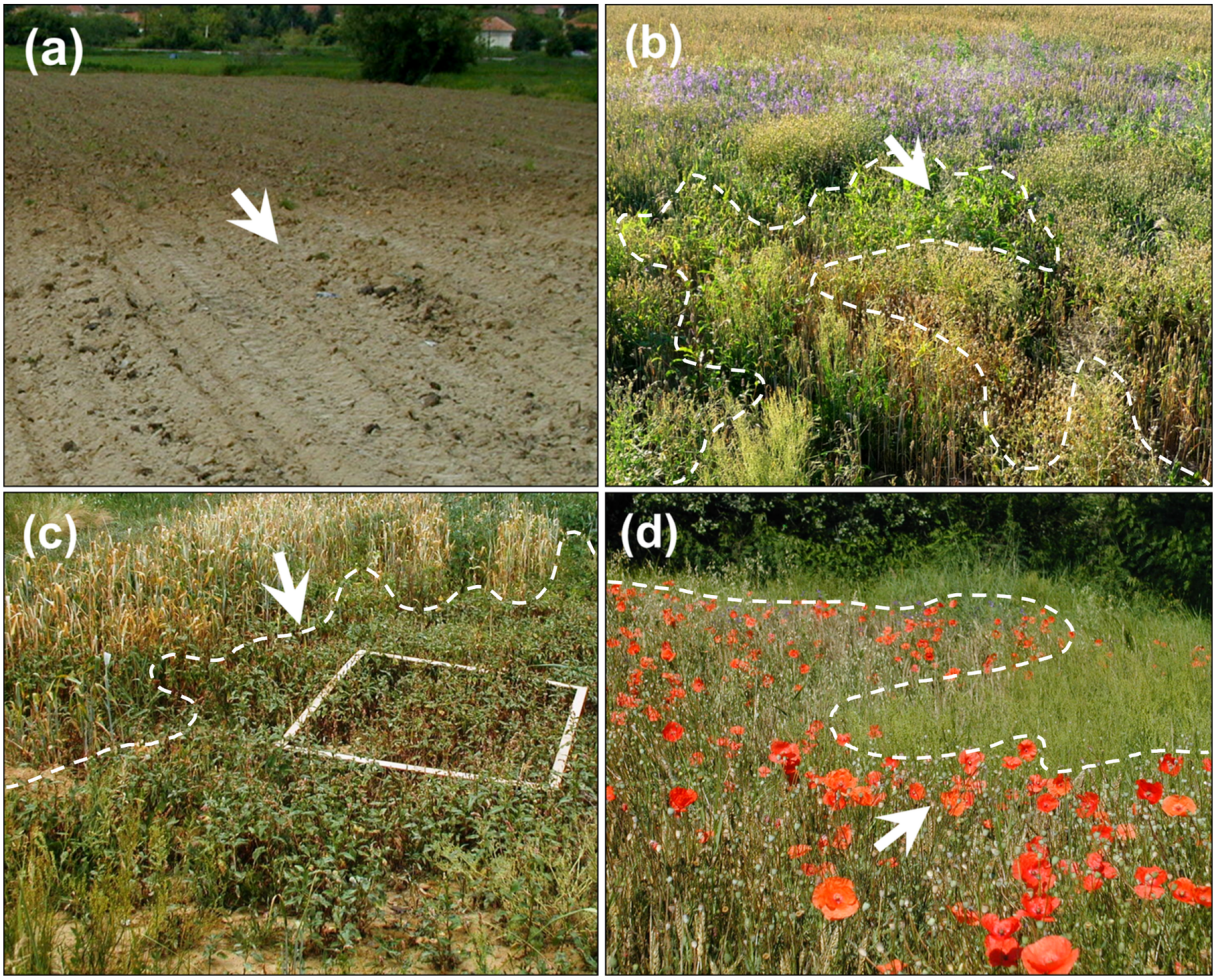
Gradients in cereal fields partially damaged by the deposition of mining waste. Arrows indicate the direction of the increasing deposition of mining waste. Soil gradient before crop emergence (a); Zone 3, Cluster B weeds, facies with *Consolida regalis* (violet flowers) (b); Zone 4, Cluster C weeds, facies with *Persicaria lapathifolia*; 1 m×1 m quadrat for biomass harvest is shown (c); Zone 3, Cluster B, facies with *Papaver rhoeas* (red flowers) (d). *Rumex acetosella*, *Agrostis capillaris* and *Persicaria lapathifolia* can be observed at the highest soil pollution levels (the “green band”, marked by dashed line; b, c and d).

**Figure 4:**
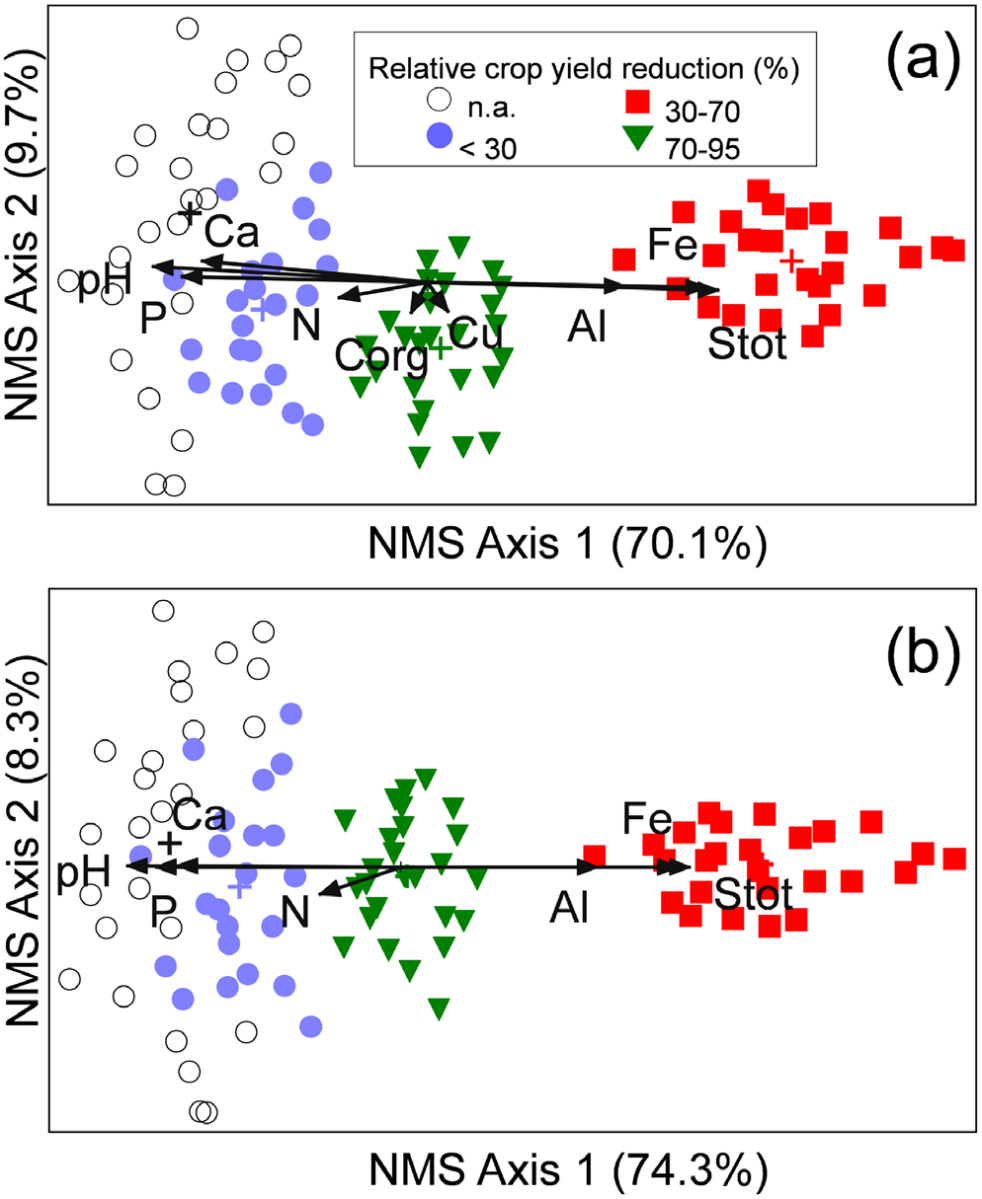
Unconstrained ordination (NMS) of weed samples along the transects in cereal fields partially damaged by mine tailings. Data matrix: 84 species (see Table S4) in 100 samples, maximal species abundances (OTV) recorded during 2 months-survey. Unrelativized OTV (a); relativized OTV (b). The values in parentheses denote the proportion of variance represented by each axis. Superimposed soil variables correlated by more than 10% with weed samples ordination are shown. The angles and lengths of the radiating lines indicate the direction and strength of relationships of the soil variables with the ordination scores. Crosses denote group centroids.

Though not a single weed species was found to be confined to one visual zone of crop growth disorders, 44 species very clearly indicate the turnover of vegetation along the soil gradient (Figure 2). Three major groups of weeds with similar response to the pollution-induced change of soil properties along the transects (Clusters A, B, and C; Figure 2) accounted for about 80% of variation in distances between weed species on the polluted fields. The succession of these three groups of weeds along the soil gradient was spatially explicit and visually very apparent over very short distances (Figure 3), commonly of less than 15 m. Accordingly, free ordination of the weed relevées showed a clear separation of vegetation along the NMS Axis 1, which corresponded to increasing pollution indicated by the increasing soil S_tot_ concentrations (Figure 4). Fifteen soil chemical parameters, which considerably varied along the transects (plant-available concentrations of Ca, Mg, K, P, B, Fe, Zn, Mn, Al, Cu; C_org_, N_tot_, S_tot_, pH, CEC) were overlaid on the ordination of weed samples; nine of them were correlated by more than 10% with the ordination scores (Figure 4, Table 3; the remaining soil properties are shown in Table S3). Little systematic variations in weed vegetation coincided with a wide gradient of soil Cu concentrations (Table 3, Figure 4). There was a high degree of multicolinearity among the soil parameters that might influence plant growth in the concentration ranges measured (Table 3): Kendall’s correlation coefficients (tau) with the NMS Axis 1 for pH, Ca, P and Al were −0.72, −0.62, −0.61 and 0.63, respectively (for the unrelativized abundances, Figure 4a; and the values were even higher for the relativized data, Figure 4b).

**Table 3:** Soil chemical properties along the pollution gradient correlated by >10% with the NMS ordination scores of weed relevées.

Parameter	Visual zones of crop growth disorders			
	1	2	3	4
	Relative yield reduction (%)			
	n.a.	<30	30–70	70–95
S_tot_ (%)	0.17±0.07 a	0.5±0.2 b	0.9±0.3 c	1.3±0.2 d
pH (in H_2_O)	7.9±0.3 a	6.9±0.3 b	5.6±0.6 c	4.6±0.5 d
C_org_ (%)	1.8±0.8 a	1.9±0.9 a	2.0±0.9 a	1.6±0.8 a
N_tot_ (%)	0.19±0.07 a	0.18±0.07 a	0.16±0.04 a	0.13±0.02 b
Ca _AAc-extr._ (mg kg^−1^)	331±36 a	283±42 b	166±33 c	139±38 c
P _AL-extr._ (mg kg^−1^)	86±14 a	75±12 a	52±20 b	28±12 c
Fe _DTPA-extr._(mg kg^−1^)	9±6 a	25±10 b	61±17 c	97±18 d
Cu _DTPA-extr._ (mg kg^−1^)	23±10 a	70±42 b	112±51 c	82±56 bc
Al _KCl-extr._ (mg kg^−1^)	0.3±0.2 a	0.7±0.6 a	27±17 b	39±25 c

Plant-available concentrations of elements (obtained by different extractions) are shown. Mean values ± SD followed by the same letter in a row are not different (*P*≤0.05).

**Figure 5:**
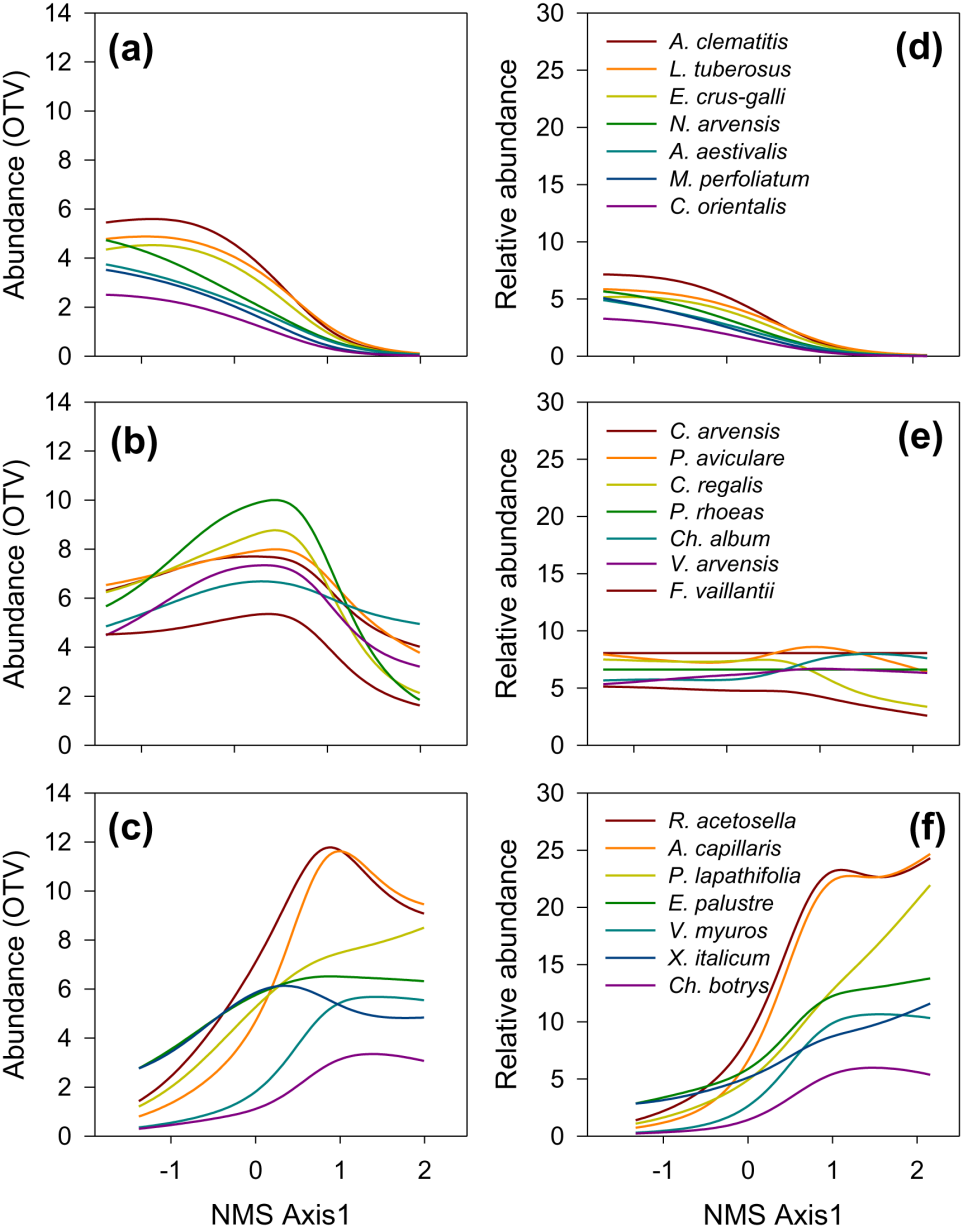
Response of the major groups of weeds to the pollution-induced soil gradient. Species envelope curves along the main ordination axis after NMS ordination of untransformed (a–c) and relativized (d–f) abundances are shown. Groups are defined after Indicator Species Analysis (IV>30%, *P*<0.01) and subsequent classification (see Figure 2). Species indicating relatively unaltered calcareous soil (a, d); species of broad valence dominant in the middle portions of the soil gradient (b, e); species indicating most severely altered, nutrient-poor acidic soils (c, f). NMS axes are scaled in standard deviations from the centroid in a normalized configuration. Relative abundance - % of the sum of OTV values in a sample.

Distinct pattern of a turnover in dominance of major weed species along the soil gradient is shown in Figure 5. Relative abundances show the trends when different competition pressure from the cereal crop is accounted for. Weeds typical for the unpolluted calcareous soils (including some rather rare, specialized calcicoles like *Nigella arvensis* and *Myagrum perfoliatum*; Figure 5a, d) were the first to disappear with increasing soil acidification by the deposited mining waste. The middle parts of the soil gradient were dominated by the common, widespread species known to have a rather broad environmental adaptations (Figure 5b, e), whereas the dominant weeds of the severely altered soils were species which did not occur on the unpolluted nearby soils of the surrounding (Figure 5c, f).

### Ecophysiological adaptations

**Figure 6:**
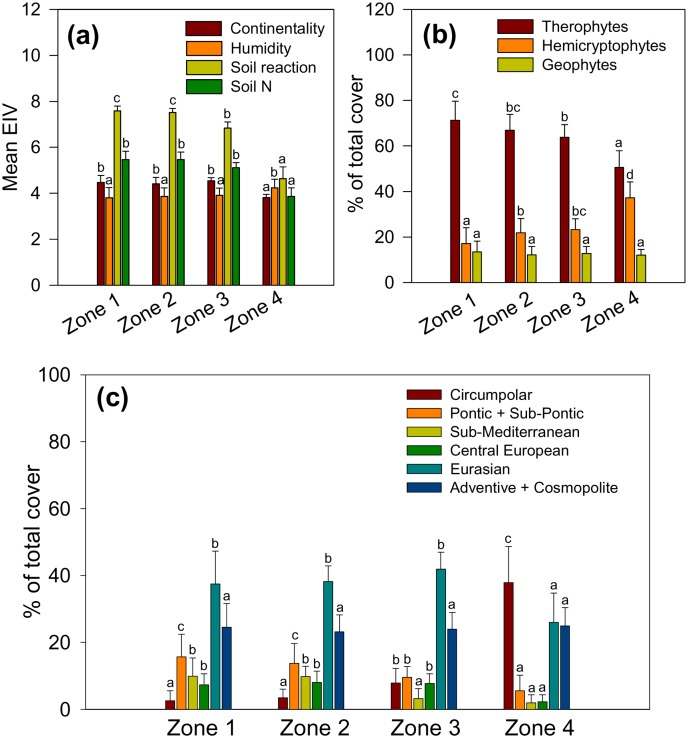
Major ecological adaptations of weed vegetation along the soil gradient. Ellenberg Indicator Values (a); life form spectra (b); chorological spectra (c). Parameters are weighted by the species cover-abundance (OTV). Weighted mean values + SD marked by the same letter in each colour-coded category are not different at *P*<0.05.

The floristic changes (Figures 3–5) made possible the continuous sustenance of weed vegetation (Figure 1) along the soil gradient from highly calcareous to highly acidic, nutrient-impoverished polluted soils. These floristic changes underpinned adaptations to significantly different habitat conditions at a field scale (Figure 6). The most prominent was a succession of different adaptations of dominant species along the soil pH gradient (Figure 6a). Under the same climatic and management conditions, in the same field, significant changes in soil properties favoured perennials with vegetative propagation (hemicryptophytes), typical of cooler and moister climates, at the expense of therophytes (Figure 6b) that normally dominate cereal weed communities. This was generally consistent with the observed decrease of the continentality index (Figure 6a). Likewise, the induced soil acidification and nutrient impoverishment underpinned the substitution of the local floral elements (most markedly of the (sub)-Pontic and sub-Mediterranean chorotype) by the adapted species of broad phytogeographical provenance (Circumpolar chorotype; Figure 6c).

**Figure 7:**
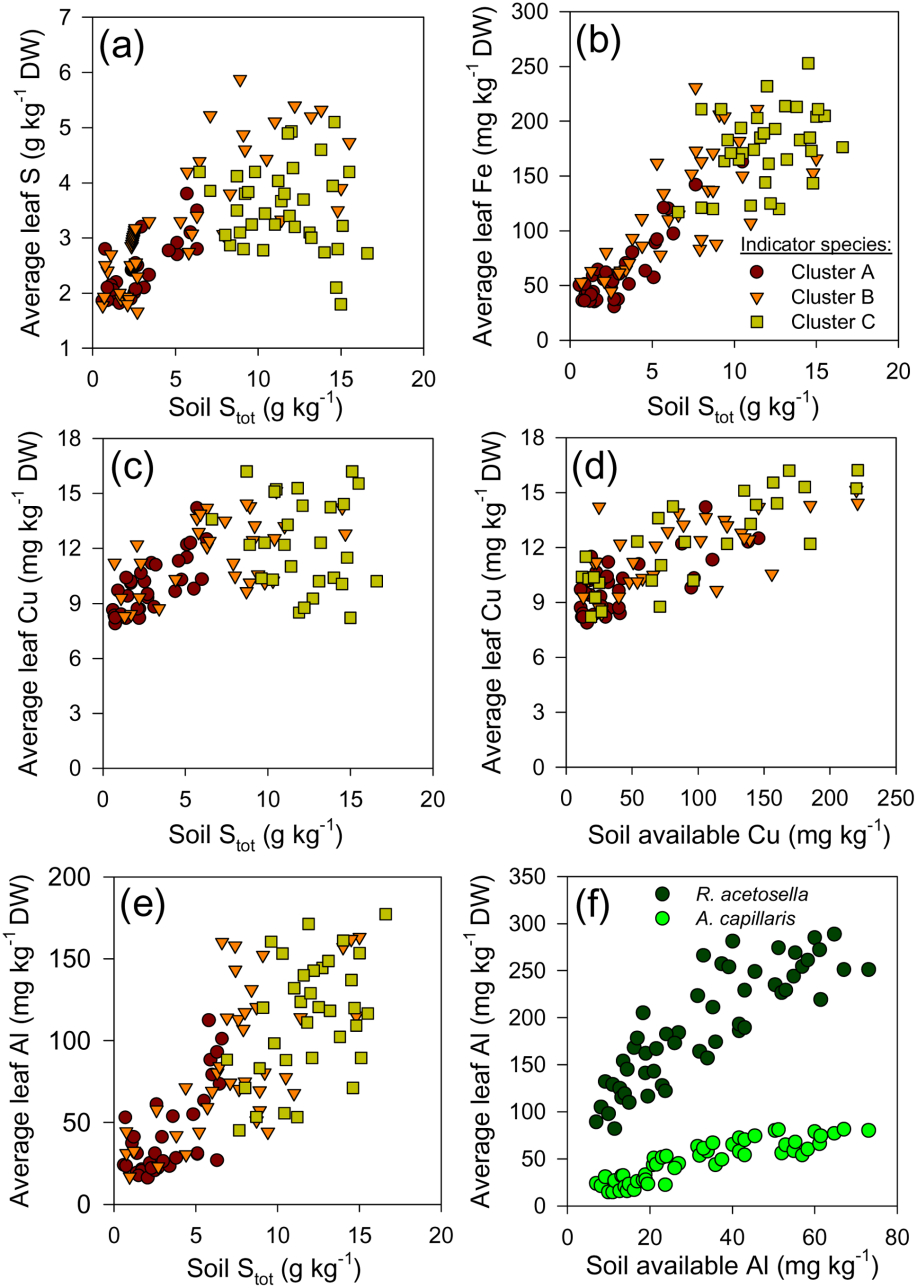
Average leaf concentrations of the major soil polluting elements (S, Fe, Cu and Al) in the three major ecological groups of species along the induced soil gradient. Leaf Al concentrations are separately shown for two key species dominant on severely degraded soils (f). Species groups are defined by Indicator Species Analysis (see Figure 2). Average group elemental concentrations were calculated by weighting the concentrations in each species by the relative proportion of a species in group biomass per m^2^. Leaves were sampled when crop was at milky ripeness phase (Z71–75).

**Figure 8:**
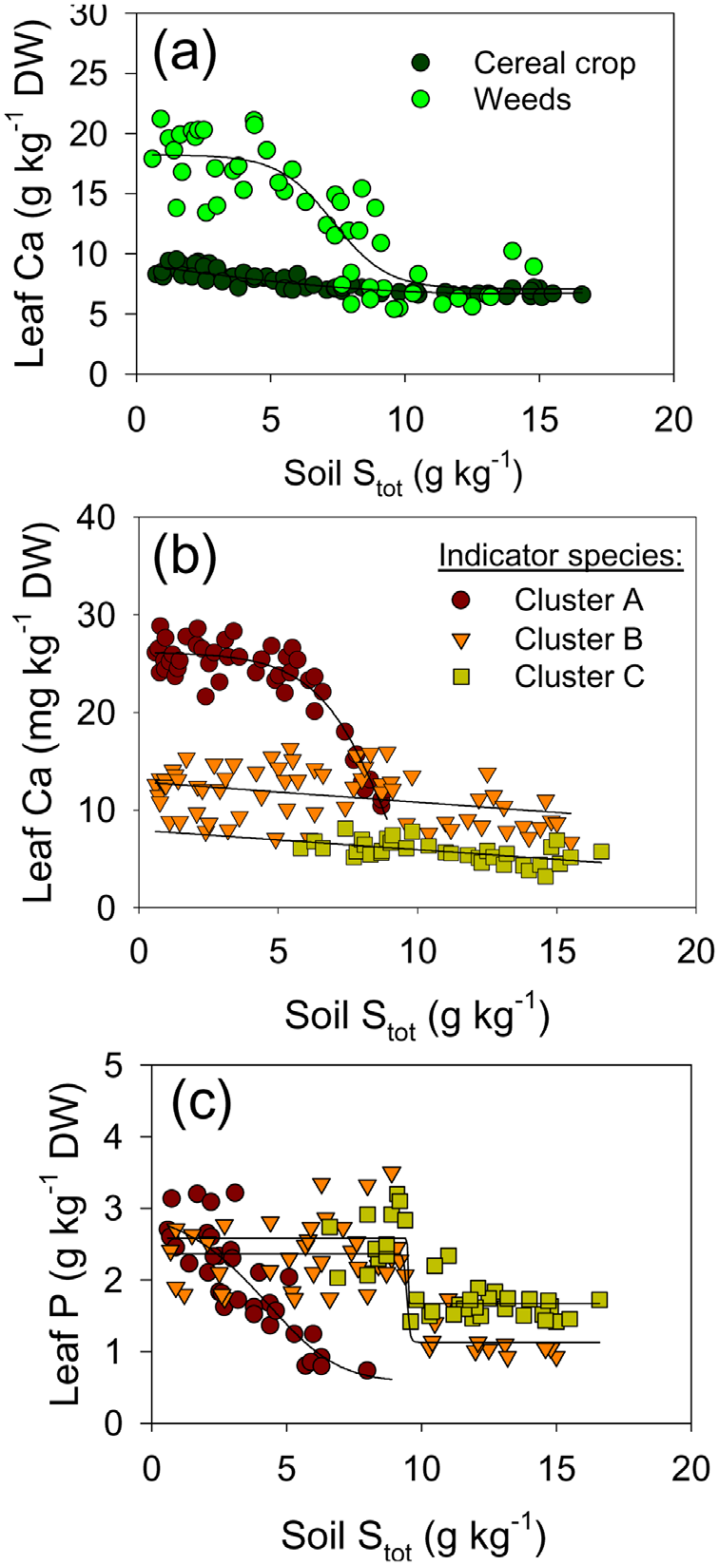
Trends in leaf concentrations of Ca and P along the pollution-induced soil gradient. Average Ca concentrations in weed vegetation and cereal crop (a); average Ca (b) and average P (c) in the three major ecological groups of species along the induced soil gradient. Species groups are defined by Indicator Species Analysis (see Figure 2). Average group elemental concentrations were calculated by weighting concentrations in each species by the relative proportion of a species in group biomass per m^2^. Leaves were sampled when crop was at milky ripeness phase (Z71–75).

The soil concentrations of mineral elements (Table 3) that were strongly correlated with the main gradients in weed vegetation (Figure 4) were differentially reflected in the foliage of weed species (Figures 7 and 8). At the weed assemblage level, the leaf concentrations of the pollutants deposited with the mining waste (S, Fe, Cu and Al) roughly reflected the increase of pollution load along the spatial transects (Figure 7), but these trends did not indicate any relation with the spatial succession of the three major groups of indicator species (see Figures 2, 3 and 5). All three ecological groups of weeds successfully maintained leaf Cu concentrations in a relatively narrow range along the soil gradient (Figure 7d). Only one species (*Chenopodium botrys*) consistently had a markedly elevated leaf Cu concentration (over 100 mg kg^−1^ DW), but it never reached an OTV>4. Moreover, the two major species adapted to the most acidified soils apparently had contrasting adaptation strategies to high extractable Al (Figure 7f). As a consequence, differences in leaf Al concentrations at the vegetation relevée level between the common weeds of a broad ecological valence (Cluster B) and weeds that strongly indicate the most severely altered soils (Cluster C) could not be detected (Figure 7e).

Contrary to the pollutants, trends in leaf concentrations of the elements whose soil concentration decreased due to waste deposition (Ca and P, Figure 8), differed among the three ecological groups of species (see Figure 5). Plant-available soil Ca decreased by 58% along the gradient (Table 3). Wheat and barley maintained a very narrow range of leaf Ca concentrations (not more than 20%-decrease in the most severely polluted soils compared to non-polluted ones, Figure 8a), whereas their growth was significantly reduced (see Figure 1). In contrast, leaf Ca concentration in weed vegetation decreased 2.5-fold along the gradient (Figure 8a). Thus, continuous weed vegetation growth along the soil gradient (Figure 1) is dependent on succession of species with differential Ca concentrations (Figure 8b). The highest leaf Ca concentrations, observed in species typical for unpolluted calcareous soils (Cluster A, see Figure 2), decreased when the complex soil pollution induced the overall disappearance of this group along the gradient (see Figure 5a, d). While the other two successive groups did not show such a severe change of leaf Ca along the soil gradient, the group of species adapted to most severely degraded soils (Cluster C) consistently maintained the lowest Ca levels (Figure 8b).

The trends in leaf P (Figure 8c) in the three species groups (defined by indicator species analysis, Figure 2) can be related to the change in abundances of these species groups along the soil pollution gradient (Figure 5). A sharp decrease of leaf P levels (below 1 g kg^−1^) in the species adapted to unpolluted calcareous soils (Cluster A, Figure 8c) was accompanied by a loss of their vitality and competitive ability (reflected in their decreased abundances; Figure 5a, d). Common species that dominated the middle portions of the pollution gradient (Cluster B) were apparently better adapted to the multiple stresses caused by soil pollution, and concomitantly they maintained leaf P concentrations (Figure 8c) and abundance (Figure 5b, e) on relatively strongly polluted soils, where Cluster A members already diminished (i.e. until Zone 4). Finally, complex adaptations of a “novel” group of species (a combination of species that did not occur as such on unpolluted nearby soils, Cluster C) to the multiple constraints on the most severely acidic soils had resulted in lowest decrease of leaf P concentrations (Figure 8c), and increased dominance (Figure 5c, f) due to cessation of competition with other species.

**Figure 9:**
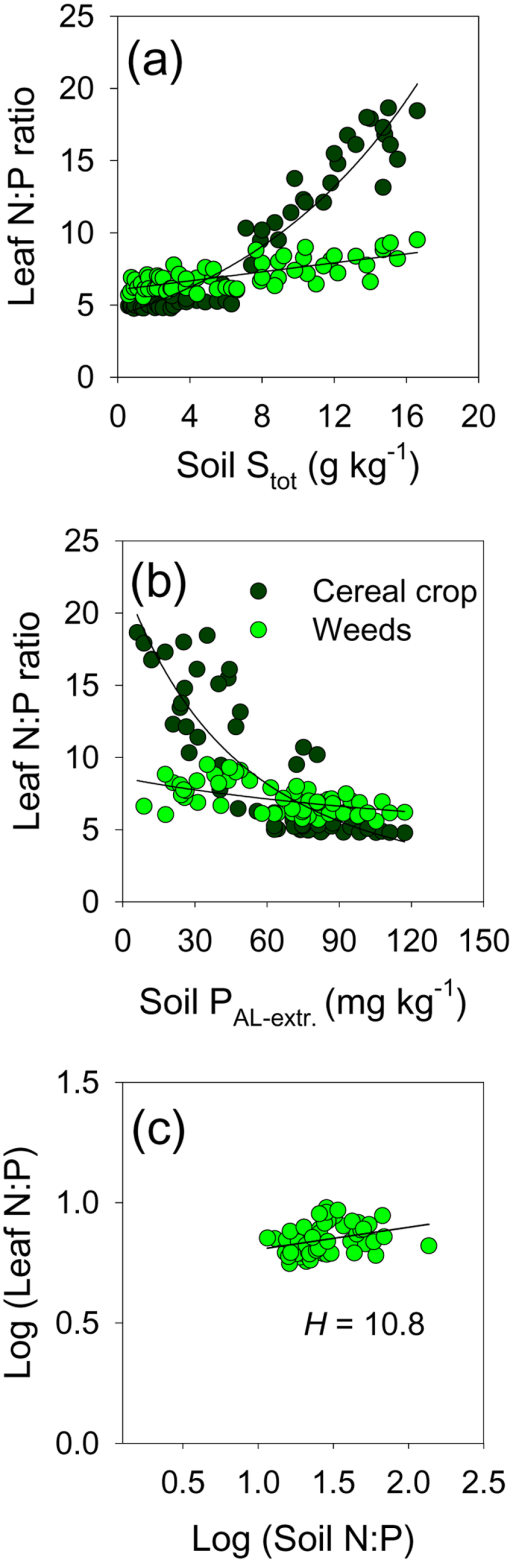
The mass ratio of N:P in weed vegetation and cereal crops along the soil pollution gradient. Leaf N:P along the complex soil gradient indicated by the pollution load (a); leaf N:P along the decreasing plant available P concentrations in polluted soils (b); biomass N:P in weed vegetation as a function of soil N:P ratio (c). *H* - regulatory coefficient, slope of the linear trend line. Leaf N and P concentrations are weighted by the relative proportion of a species in total biomass per m^2^, sampled when crop was at milky ripeness phase (Z71–75).

Finally, the sustenance of weed growth (Figure 1), accompanied by the remarkable change in floristic composition of weed vegetation (Figures 2–5) was underpinned by the maintenance of the N:P ratio in the biomass of weeds along the soil pollution gradient, while this ratio had not been sustained in the crop biomass (Figure 9a). Moreover, the same pattern of change of the biomass N:P ratio between the weeds and the cereal crops was observed along the gradient of plant-available P in the polluted soils (Figure 9b). Figure 9c shows that the weed biomass N:P ratio was only very weakly influenced by the external supply of N and P (in the range of soil N:P values measured in this study). This was also indicated by a high value of the regulatory coefficient *H* of 10.8 (Figure 9c). The value of the regulatory coefficient *H* for cereal crops (wheat and barley) was much lower (1.56, not shown).

## Discussion

Causality in environment-vegetation relationship in studies based on observational analysis like the one reported here is difficult to prove, and it is widely denigrated as mere correlation [28]. The present study has shown that exceptionally strong gradients in weed vegetation (accounting for over 70% of the observed variation) were caused by a complex pollution-induced soil gradient (Figure 4); all the other plausible environmental factors were kept constant over short transects investigated (on average less than 15 m), and differential competition pressures from the cereal crop were accounted for by relativization transformation (Figures 2, 4b and 5d–f). Broadly speaking, change in soil properties from calcareous (pH>7.5) to strongly acidic, leached, nutrient-poor soils (average pH 4.6, Table 3) was accompanied by a change of species adaptations which indicate different habitat conditions (Figure 6) as well as by a shift from the dominance of “calcicole” (Cluster A, Figure 2) species to “calcifuges” (Cluster C, see also Figures 4 and 5). These findings are in accordance with a general substitution of weed species belonging to Caucalidion lappulae Tx. 1950 alliance of warmer, drier, calcareous soils by species of the Aperetalia spicae-venti Tx. and Tx. 1960 order in moister, cooler climates and acidic soils which however takes place over a large geographic gradient [29]. The species composition of the analyzed weed vegetation (Table S4) was significantly affected by (a) a rather low level of field management, and (b) surrounding vegetation matrix of predominantly fallow land in different successional stages. The former had enabled the maintenance of some species that have become rare in more-intensively managed agricultural landscapes, while the latter contributed to a rather high proportion of non-segetal species. As a result, a high proportion of species known as pseudometallophytes (as defined by [30]; Clusters B and C, Figure 2, see also Table S4) constituted an important response of weed vegetation to soil pollution gradient.

While species co-occurrence is a result of two simultaneous processes with contrasting effects: “habitat filtering” and “limiting similarity” [31], in severe environments like post-mining sites communities are assembled primarily by environmental filtering [11]. The assemblages on the most severely affected soils (Zone 4) were characterized by: (a) dominance of few typical acidophytic species (*Apera spica-venti*, *Vicia hirsuta*, *Rumex acetosella*, and *Agrostis capillaris*, in Cluster C, Figure 2), which are not sustained by the regional climate (semiarid conditions, Figure S1), carstic geology and calcareous soils and xerothermic natural vegetation; (b) the lowest variability of weed relevées along the soil gradient (Table 2, Figure 4), and the strongest association among the dominant species (Cluster C, Figure 2); and c) an unlikely combination of species that normally do not occur together (for example, *Agrostis capillaris* is not a cereal weed; *Chenopodium botrys* is also not a segetal species, and is not found on moist, acid soils in cool areas; *Vulpia myuros* and *Xanthium italicum* vs. *Equisetum palustre* and *Persicaria lapathifolia* have contrasting preferences for humidity). These findings clearly demonstrate the key effect of severe abiotic filtering on weed assembling. Firstly, small-scale soil factors (Zone 4, see Table 3) overrode the hierarchically higher filters of regional flora and climate which normally have a primary effect on plant communities [32]. Likewise, the increasing differences between weed communities along the soil pollution gradient (Figure 4) are in accordance with the reported phenomenon that increasing intensity of abiotic constraint leads to increasing differences of assembled communities from the regional species pool [33]. Secondly, intensification of habitat constraints has been shown to increase aggregation of dominant species [33], [34], and in particular it can increase the degree of association of specialist weed species at the field scale [6]. The dominant species *Agrostis capillaris*, *Rumex acetosella* and, less apparently *Persicaria lapathifolia* (see Figure 5c, f) are frequently found pioneer colonizers on mining-affected, acidic and nutrient-poor substrates, which are often also characterised by high concentrations of plant-available heavy metals [35], [36]; likewise, they possess certain tolerance to flooding, what is altogether a wide range of adaptations not usually encountered in nature. Shared ecological tolerance (i.e. trait convergence) is a common feature of communities assembled primarily by environmental filtering [37]. Thus, specific adaptations required to survive under complex, anthropogenically induced soil constraints are apparently the main biological reason underlying the consistent co-occurrence of a small number of dominant species in Zone 4. Thirdly, non-historic species combinations due to strong environmental change are the key feature of the novel ecosystem concept [13], [14]. The main functions of novel ecosystems are often preserved although biotic composition is strongly altered [15]; the underlying biological mechanisms leading to a particular novel species composition are however widely unknown.

While it was possible to demonstrate the response of weed vegetation (Figures 2–5) to a complex soil gradient (Table 3; see also Table S3), the causal hierarchy of specific soil factors has remained in domain of black box. For wheat and barley on the same soils, growth reduction occurred due to a myriad of possible interactions among the major soil constraints (P deficiency, low pH, excessive concentrations of Al and Cu), as discussed previously [16], [17]. In the present study, the measured response parameters of the vegetation (biomass, floristic composition and leaf mineral composition) could not show the type of adaptations to all the significant pollution-induced soil constraints (shown in Figure 4 and Table 3). For instance, while soil pH is a well-known major factor that underpins vegetation response [29], our methodology could indicate only complex adaptations to acidic soils along the pollution gradient (Ellenberg indicator value for soil reaction; Figure 6a). Likewise, we did not detect any trend in leaf concentrations of elements deposited by soil pollution (Figure 7) which could be related to the induced gradient in weed vegetation. Moreover, the relatively high concentrations of available soil Cu (Table 3; for example, concentrations of only about 30 mg kg^−1^ were toxic to cereals; [38]) were not found to be significantly correlated with any observed pattern in weed vegetation (Figure 4). Trends in vegetation response to the elevated soil Cu concentrations were, *inter alia*, obscured by the importance of pseudometallophytes, species that commonly possess certain tolerance to metals, mostly based on exclusion [30], what was reflected in the narrow range of leaf Cu concentrations (Figure 7d). Even though adaptations to metal-contaminated soil have been extensively investigated at the level of individual species, the effects on vegetation have seldom been examined [39]. An overriding effect of metals on the vegetation patterns in metal-contaminated soils is usually assumed; nevertheless, this conclusion has commonly been reached without concomitant consideration of the plant availability of the major nutrients in these soils [40]. On the other hand, several earlier reports have pointed out nutrient deficiency and in particular P deficiency as a key factor that affects the structure of vegetation on soils polluted by metals [35], [41]–[43].

Contrary to the elements whose soil concentrations were anthropogenically increased, we did show clear connection between soil-induced species turnover and the underlying functional adaptations to decreasing availability of Ca and P, as well as a strong capacity of vegetation to maintain biomass N:P ratio along the gradient. The pollution-induced decrease of soil Ca concentrations (Table 3), highly correlated with the changes in weed vegetation (Figure 4), was accompanied by a clear spatial succession of species with differential leaf Ca concentrations (Figure 8b). In their natural environments, plants adapted to calcareous soils (“calcicoles”) commonly have higher shoot Ca than plants adapted to acidic soils (“calcifuges”); calcifuges likely have lower Ca requirements [8], [44]. These two functional vegetation types possess widely differing key physiological adaptations to other particular soil constraints beside Ca levels (low pH, P deficiency, Al-, Mn- and Fe-toxicity on acidic, and P- and micronutrient deficiency in calcareous soils [45]), and their occurrence is further complexly affected by climatic gradient and some other evolutionary and historic processes [46]. Next, soil-induced species turnover along the gradient (Figures 3 and 4) can be related to a spatial sequence of ecological groups which successively achieve both dominance (see Figure 5) and the highest leaf P concentration (Figure 8c). A large scale-survey in UK [8] showed that “normal” P leaf concentrations in the species encountered also in our study (in Clusters B and C, Figure 2) ranged between 2 and 4 g kg^−1^, what was comparable to the leaf P status at the “peak abundance” of each ecological group in our study (see Figures 5 and 8c). The floristic changes induced by a complex soil pollution gradient conveyed the plasticity of the vegetation response to the external P supply; the available soil P decreased more than three times (Table 3), while weed vegetation maintained a rather low variation in leaf P (decrease by 40%, calculated from Figure 8c). Finally, the N:P ratio in weed biomass varied in a rather narrow range (Figure 9). At the level of weed vegetation in the damaged fields, the value of the regulatory coefficient *H* was extremely high (Figure 9c). So far, *H* reported for individual species was below 5 [27], [47].

Leaf concentrations of mineral elements have very different potential to be used as true species traits. The strength of homeostatic regulation of concentration of mineral elements in plants in response to substrate widely differs among elements as well as among plant species and functional groups, so that meaningful results might be uncertain to obtain [8], [9]. Moreover, for most weed species critical deficiency and toxicity concentrations of mineral elements in leaves have not been established [48]. For instance, leaf Ca concentrations (under optimal conditions) vary considerably across plant species, more than P concentrations and much more than N concentrations [48]. Three physiotypes for Ca nutrition with intrinsically different leaf Ca levels have been described, and Ca deficiency is rarely encountered in nature [44]. Therefore, though a spatial succession of calicole by calcifuge species along the gradient of decreasing soil Ca levels was demonstrated (Figures 5 and 8b; Table 3), it remained unclear whether very different leaf Ca levels (2.5-fold, Figure 8b) along the induced vegetation gradient were a trait required *per se* by the imposed environmental filters. Furthermore, the mass N:P ratio as an indicator of nutrient limitation and nutrient cycling in ecosystems has extensively been used to study vegetation change [47], though controversies on its reliability exist (as discussed by [49]). Interspecific variation of N:P ratio is primarily caused by varying leaf P concentrations [47]. In our study soil N levels were rather low along the whole gradient (Table 3) and we did not detect significant change of leaf N along the gradient (not shown). The reason might be the internal N-use efficiency which enables plants to maintain the critical N tissue concentrations by regulating growth rate, e.g. [50]; hence, leaf N concentrations below the critical level are not likely to be recorded in viable spontaneously growing plants. Finally, P has previously been indicated as the only mineral element whose leaf concentrations (closely correlated with leaf N) could be considered a legitimate species trait [8]. Plants have different strategies to cope with P deficiency which occurs on both calcareous and acidic soils [51]. Thus, nutrient acquisition and utilization strategies might have changed with species composition along the soil gradient (as demonstrated by [52]). The present study showed that the final effect of a complex of physiological adaptations to changing soil constraints, conveyed by the induced species turnover, was homeostasis of leaf P and N:P ratio in weed vegetation. Environmental filtering (primarily by severe soil constraints), as the key assembly process, determined weed species co-occurrence by filtering out species with the same adaptations to the dominant constraint along the gradient (as shown by [37]); the sum effect of these adaptations was indicated by leaf nutrient concentrations (Figures 8 and 9).

A very high variability in species composition induced by strong soil gradient was accompanied by low variability of both leaf P concentrations (Figure 8c) and biomass N:P ratio (Figure 9). Thus, under severe environmental conditions and using leaf nutrient concentrations as species traits, this study supports the core findings of a manipulative experiment of [53] that environmental filtering can cause the composition of traits to converge even while species composition diverges. This divergence of species composition led to novel associations with unusual species combinations but only weakly affected biomass production (Figure 1), supporting a wider notion on functionality of novel ecosystems [15]. Finally, novel ecosystems, recognized to emerge due to anthropogenic disturbance, have been studied mostly from the perspective of management and restoration [13]–[15], [54]; this study indicated one of the underlying physiological drivers of “novelty”, confirming the intriguing potential of accidental experiment sites for purely ecological research.

## Conclusions

Using cereal weeds as a model system on an “accidental experiment” location with a distinct gradient of polluted soil over short distances (less than 15 m), we demonstrated links between gradients in soil properties and floristic composition of spontaneous vegetation. The response of vegetation to a gradient from highly calcareous to highly acidic, nutrient-poor and Cu-enriched soils included a substitution of calcicoles by calcifuges, and an increase in abundance of species known as pseudometallophytes, with preferences for Atlantic climate, broad geographical distribution, hemicryptophytic life form, adapted to low-nutrient and acidic soils, with lower concentrations of Ca, and very narrow range of Cu concentrations in leaves. The trends of abundance of the successive ecological groups of indicator species along the soil gradient underpinned a clear functional response to mineral stress, systematically reflected in the maintenance of leaf P concentrations and strong homeostasis in biomass N:P ratio. These findings help to understand the driving forces of assembly processes leading to unusual, novel combinations of species which are commonly observed as a consequence of strong environmental filtering, as for instance on sites affected by industrial activities.

## Supporting Information

Figure S1 **Climatic conditions of the research area.**
(DOCX)

Table S1 **Weed assemblages significantly differ among the visual zones of crop growth on polluted soils (Multi-Response Permutation Procedure test).**
(DOCX)

Table S2 **Floristic difference (presence/absence data, observed during the 2-months survey) of cereal weeds of among the visual zones of crop growth on polluted soils.**
(DOCX)

Table S3 **Soil chemical properties not shown in Table 4.**
(DOCX)

Table S4 **List of weed species included in the analysis.**
(DOCX)
