# Supplementary material for: Assembly Processes under Severe Abiotic Filtering: Adaptation Mechanisms of Weed Vegetation to the Gradient of Soil Constraints
Source: PLoS One. 2014 Dec 4;9(12):e114290. doi: 10.1371/journal.pone.0114290 (PMC4256224; doi:10.1371/journal.pone.0114290)
Supplement: Figure S1 — Climatic conditions of the research area. (DOCX) [file pone.0114290.s001.docx]

**
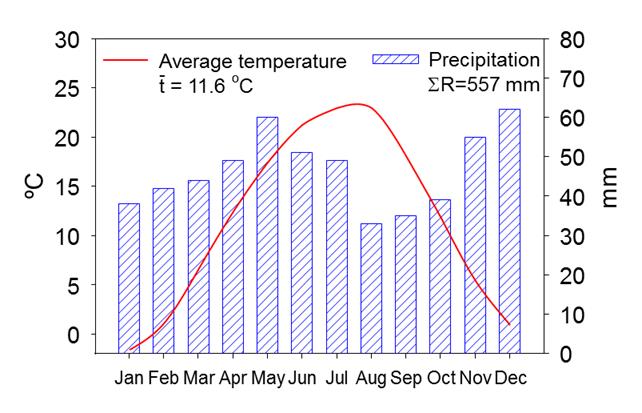
**

**Figure S1**: Climatic conditions of the research area. Climate diagram according to Walter: t – average annual temperature; ΣR – total annual precipitation; data for the Meteorological Station Negotin (about 25 km from the research site), 1961-1999.
