# Supplementary material for: Assembly Processes under Severe Abiotic Filtering: Adaptation Mechanisms of Weed Vegetation to the Gradient of Soil Constraints
Source: PLoS One. 2014 Dec 4;9(12):e114290. doi: 10.1371/journal.pone.0114290 (PMC4256224; doi:10.1371/journal.pone.0114290)
Supplement: Table S1 — Weed assemblages significantly differ among the visual zones of crop growth on polluted soils (Multi-Response Permutation Procedure test). (DOCX) [file pone.0114290.s002.docx]

**Table S1**: Multi-Response Permutation Procedure test (Sørensen distance) shows that weed assemblages significantly differ among the visual zones of crop growth on polluted soils.

|  | Absolute abundance | | Relative abundance | |
| --- | --- | --- | --- | --- |
| Overall comparison | A = 0.236, P < 10^-8^ | | A = 0.214, P < 10^-8^ | |
| Multiple comparisons among the zones | T | A | T | A |
| 1 vs 2 | -7.10 | 0.022 | -4.66 | 0.014 |
| 1 vs 3 | -29.26 | 0.122 | -25.95 | 0.091 |
| 1 vs 4 | -33.55 | 0.252 | -33.50 | 0.239 |
| 2 vs 3 | -21.26 | 0.079 | -19.34 | 0.067 |
| 2 vs 4 | -31.12 | 0.274 | -30.84 | 0.253 |
| 3 vs 4 | -33.14 | 0.245 | -32.38 | 0.222 |

A: chance-corrected within-group agreement; it describes the homogeneity within the zones compared to the random expectation T: statistic describes the separation between the samples in different zones; the more negative is T, the stronger is the separation.
