# Supplementary material for: Assembly Processes under Severe Abiotic Filtering: Adaptation Mechanisms of Weed Vegetation to the Gradient of Soil Constraints
Source: PLoS One. 2014 Dec 4;9(12):e114290. doi: 10.1371/journal.pone.0114290 (PMC4256224; doi:10.1371/journal.pone.0114290)
Supplement: Table S2 — Floristic difference (presence/absence data, observed during the 2-months survey) of cereal weeds of among the visual zones of crop growth on polluted soils. (DOCX) [file pone.0114290.s003.docx]

**Table S2**: The change in species composition (presence/absence data) of cereal weeds observed during the 2-months survey of 100 samples along the soil gradient.

|  | Shared species | |
| --- | --- | --- |
| Visual zones in polluted cereal fields | Number | % of total in both zones |
| 1 and 2 | 76 | 95.0 |
| 1 and 3 | 73 | 93.4 |
| 1 and 4 | 53 | 63.1 |
| 2 and 3 | 76 | 89.4 |
| 2 and 4 | 56 | 65.9 |
| 3 and 4 | 60 | 73.2 |
