# Supplementary material for: Assembly Processes under Severe Abiotic Filtering: Adaptation Mechanisms of Weed Vegetation to the Gradient of Soil Constraints
Source: PLoS One. 2014 Dec 4;9(12):e114290. doi: 10.1371/journal.pone.0114290 (PMC4256224; doi:10.1371/journal.pone.0114290)
Supplement: Table S3 — Soil chemical properties not shown in Table 4. (DOCX) [file pone.0114290.s004.docx]

**Table S3**: Soil chemical properties along the pollution gradient correlated by < 10% with the NMS ordination scores of weed relevées. Plant available concentrations of elements (obtained by different extractions) are shown. Mean values ± SD followed by the same letter in a row are not different (*P* ≤ 0.05).

| Parameter | Visual zones of crop growth disorders | | | |
| --- | --- | --- | --- | --- |
|  | 1 | 2 | 3 | 4 |
|  | Relative yield reduction (%) | | | |
|  | n.a. | < 30 | 30-70 | 70-95 |
| CEC (meq 100g^-1^) | 19.6 ± 1.6 a | 17.7 ± 1.5 b | 16.1 ± 1.5 c | 12.6 ± 2.4 d |
| Mg _AAc-extr._ (mg kg^-1^) | 21 ± 2 a | 19 ± 2 b | 16 ± 2 c | 15 ± 3 c |
| K _AL-extr._ (mg kg^-1^) | 207 ± 31 a | 173 ± 25 ab | 164 ± 16 b | 143 ± 41 b |
| Zn _DTPA-extr._(mg kg^-1^) | 2 ± 2 ab | 3 ± 2 ab | 3 ± 2 b | 2 ± 1 a |
| Mn _DTPA-extr._ (mg kg^-1^) | 15 ± 3 a | 13 ± 3 a | 14 ± 7 a | 11 ± 6a |
| B _hot water -extr_ (mg kg^-1^) | 0.9 ± 0.1 a | 0.7 ± 0.3 a | 0.42 ± 0.08 b | 0.35 ± 0.09 b |
