# Supplementary material for: Assembly Processes under Severe Abiotic Filtering: Adaptation Mechanisms of Weed Vegetation to the Gradient of Soil Constraints
Source: PLoS One. 2014 Dec 4;9(12):e114290. doi: 10.1371/journal.pone.0114290 (PMC4256224; doi:10.1371/journal.pone.0114290)
Supplement: Table S4 — List of weed species included in the analysis. (DOCX) [file pone.0114290.s005.docx]

**Table S4**: Weed list (84 species included in the analyses). Of all the species recorded during the 2-month survey only those with overall frequency >10% or, frequency in at least one sampling zone ≥ 20%, are included. Nomenclature follows the W3 TROPICOS database of the Missouri Botanical Garden.

| Species | Reference | Family |
| --- | --- | --- |
| *Adonis aestivalis* | L. | Ranunculaceae |
| *Aethusa cynapium* | L. | Apiaceae |
| *Agropyrum intermedium* | (Host) P. Beuv. | Poaceae |
| *Agrostemma githago* | L. | Caryophyllaceae |
| *Agrostis capillaris* | L. | Poaceae |
| *Ajuga chamaepitys* | Schreb. | Lamiaceae |
| *Amaranthus retroflexus* | L. | Amaranthaceae |
| *Anagallis arvensis* | L. | Primulaceae |
| *Anchusa arvensis* | M. Bieb. | Boraginaceae |
| *Anthemis arvensis* | L. | Asteraceae |
| *Apera spica-venti* | (L.) P. (Beauv). | Poaceae |
| *Aristolochia clematitis* | L. | Aristolochiaceae |
| *Artemisia vulgaris* | L. | Asteraceae |
| *Avena fatua* | L. | Poaceae |
| *Barbarea vulgaris* | W. T. Aiton | Brassicaceae |
| *Berteroa incana* | (L.) DC. | Brassicaceae |
| *Bifora radians* | M. Bieb. | Apiaceae |
| *Bilderdykia convolvulus* | (L.) Dumort. | Polygonaceae |
| *Bromus sterilis* | L. | Poaceae |
| *Caucalis platycarpos* | L. | Apiaceae |
| *Centaurea cyanus* | L. | Asteraceae |
| *Centaurea jacea* | L. | Asteraceae |
| *Chenopodium album* | L. | Amaranthaceae |
| *Chenopodium botrys* | L. | Amaranthaceae |
| *Chondrilla juncea* | L. | Asteraceae |
| *Cichorium intybus* | L. | Asteraceae |
| *Cirsium arvense* | (L.) Scop. | Asteraceae |
| *Conringia orientalis* | (L.) C. Presl. | Brassicaceae |
| *Consolida regalis* | Gray | Ranunculaceae |
| *Convolvulus arvensis* | L. | Convolvulaceae |
| *Crepis tectorum* | L. | Asteraceae |
| *Daucus carota* | L. | Apiaceae |
| *Echinochloa crus-galli* | (L.) P. (Beauv). | Poaceae |
| *Equisetum palustre* | L. | Equisetaceae |
| *Erigeron canadensis* | L. | Asteraceae |
| *Eryngium campestre* | L. | Apiaceae |
| *Euphorbia falcata* | L. | Euphorbiaceae |
| *Euphorbia helioscopia* | L. | Euphorbiaceae |
| *Fumaria vaillantii* | Loisel. | Papaveraceae |
| *Galium aparine* | L. | Rubiaceae |
| *Kickxia spuria* | (L.) Dumort. | Plantaginaceae |
| *Lactuca serriola* | L. | Asteraceae |
| *Lamium purpureum* | L. | Lamiaceae |
| Species | Reference | Family |
| *Lapsana communis* | L. | Asteraceae |
| *Lathyrus aphaca* | L. | Fabaceae |
| *Lathyrus nissolia* | L. | Fabaceae |
| *Lathyrus tuberosus* | L. | Fabaceae |
| *Lepidium draba* | L. | Brassicaceae |
| *Linaria vulgaris* | Mill. | Plantaginaceae |
| *Mentha arvensis* | L. | Lamiaceae |
| *Myagrum perfoliatum* | L. | Brassicaceae |
| *Nigella arvensis* | L. | Ranunculaceae |
| *Papaver rhoeas* | L. | Papaveraceae |
| *Persicaria lapathifolia* | (L.) Gray | Polygonaceae |
| *Persicaria maculosa* | Gray | Polygonaceae |
| *Plantago lanceolata* | L. | Plantaginaceae |
| *Poa pratensis* | L. | Poaceae |
| *Polygonum aviculare* | L. | Polygonaceae |
| *Ranunculus arvensis* | L. | Ranunculaceae |
| *Rorippa sylvestris* | (L.) Besser | Brassicaceae |
| *Rumex acetosella* | L. | Polygonaceae |
| *Rumex crispus* | L. | Polygonaceae |
| *Scandix pecten-veneris* | L. | Apiaceae |
| *Senecio rupestris* | Waldst. | Asteraceae |
| *Setaria glauca* | (L.) P. Beauv. | Poaceae |
| *Silene latifolia* subsp. *alba* | (Mill.) Greuter & Burdet | Caryophyllaceae |
| *Silene noctiflora* | L. | Caryophyllaceae |
| *Sinapis arvensis* | L. | Brassicaceae |
| *Solanum nigrum* | L. | Solanaceae |
| *Sonchus oleraceus* | L. | Asteraceae |
| *Stachys annua* | L. | Lamiaceae |
| *Thymelaea passerina* | (L.) Coss. & Germ. | Thymelaeaceae |
| *Torilis arvensis* | (Huds.) Link | Apiaceae |
| *Vaccaria pyramidata* | Medik. | Caryophyllaceae |
| *Valerianella rimosa* | Bast | Caprifoliaceae |
| *Verbena officinalis* | L. | Verbenaceae |
| *Veronica persica* | Poir. | Plantaginaceae |
| *Veronica polita* | Fr. | Plantaginaceae |
| *Vicia cracca* | L. | Fabaceae |
| *Vicia grandiflora* | Scop. | Fabaceae |
| *Vicia hirsuta* | (L.) Gray | Fabaceae |
| *Viola arvensis* | Murray | Violaceae |
| *Vulpia myuros* | (L.) C.C. Gmel. | Poaceae |
| *Xanthium italicum* | Moretti | Asteraceae |
| *Xeranthemum annuum* | L. | Asteraceae |
